# Balancing between reality, ideality, and equity: critical reflections from recruiting key informants for qualitative health research

**DOI:** 10.1186/s12874-024-02403-2

**Published:** 2024-11-12

**Authors:** Hanna Luetke Lanfer, Sarah Krawiec, Miriam Schierenbeck, Victoria Touzel, Doreen Reifegerste

**Affiliations:** School of Public Health, Bielefeld University, Universitaetsstrasse 25, 33615 Bielefeld, Germany

**Keywords:** Key informant interviews, Qualitative Health Research, Intersectionality, Recruitment strategies, Equity

## Abstract

**Background:**

Key informant interviews (KII) are a widely used method in qualitative health research to gain in-depth insights from individuals with specialized knowledge, experience, or access that is crucial to the research topic. However, there is growing criticism regarding how the selection of key informants is insufficiently described in research. This opacity is problematic as the authority and knowledge of key informants may be given undue weight in research findings, potentially overshadowing other non-expert samples. The resulting imbalance in representation can lead to favoring certain viewpoints while marginalizing others, and thereby reinforcing existing inequities.

**Methods:**

Using our KII study as an example, we demonstrate how we initially composed an ideal sample based on theoretical considerations and subsequently operationalized it in the field. We employed a selective recruitment strategy informed by intersectional theory, targeting physicians with migration backgrounds from Middle Eastern countries for a study on cancer prevention and screening. Our recruitment process combined direct methods, including database searches and email outreach, with indirect methods like snowball sampling and engagement with multipliers. The recruitment strategy was iterative, allowing for ongoing assessment and adaptation to ensure a diverse and representative sample.

**Results:**

The KII study successfully recruited 21 physicians with diverse social categories, including different genders, migration backgrounds, language skills, and medical specialties. Direct recruitment was more effective than indirect methods and allowed for greater control in reaching out to specific subsamples. It highlights the importance of flexible and persistent recruitment strategies to achieve the desired sample.

**Conclusions:**

This KII study underscores the interplay between methodological ideals and the practical realities of recruiting a diverse, carefully composed sample of key informants in health research. Our intersectional approach aimed to ensure equitable representation by considering power dynamics and refining recruitment strategies, while balancing the challenges of real-world fieldwork-such as engaging busy physicians with specific recruitment criteria-with practical adaptability. Our KII study emphasizes the need for ongoing reflexivity to balance ideality and equity with practical feasibility.

**Supplementary Information:**

The online version contains supplementary material available at 10.1186/s12874-024-02403-2.

## Introduction

Key informant interviews (KII) are a frequently used method to gain in-depth insights into specific health-related issues in qualitative health and other social science research. Key informants are individuals who are viewed as possessing specialized knowledge, experience, or access that is crucial to the research topic [1, 2]. Engaging with them may be vital for accessing insider knowledge, particularly on sensitive topics that might not emerge in focus group discussions [3]. Moreover, key informants frequently serve as gateways to broader communities, helping researchers identify additional participants or facilitating community access [4, 5].

At the same time, there is a growing critique regarding how processes to select key informants are described, or rather not described in research and the potential power imbalances these processes can perpetuate [6, 7]. Unlike regular, non-expert participants (hereafter referred to as “regular participants”) whose socio-economic data and demographic details are often documented in great detail, the selection criteria for key informants tend to remain rather vague or are omitted [8]. This opacity is problematic as the authority and knowledge of key informants may be disproportionately weighted in research findings, potentially overshadowing other voices [8–10]. The resulting imbalance in representation can skew the production of knowledge, privileging certain perspectives while marginalizing others, thereby exacerbating existing inequities [11].

While key informants are important for their insights, it is crucial to approach the recruitment of key informants with a focus on both intersectionality and transparency [12, 13]. Given that the process of knowledge production and representation in qualitative research is deeply influenced by the social and cultural contexts in which it occurs, the insights provided by key informants are shaped by the informants’ specific positions within these contexts and need to be even more reflected, discussed and documented than those of regular participants [7, 14], especially in research areas like health, where the implications of findings are directly tied to real-world policies and practices [15].

Yet, even as we strive for methodological ideals, the realities of fieldwork often present operational challenges in reaching the desired sound, diverse, and representative sample. For example, in our KII study on the barriers to cancer screening and prevention among first-generation migrants from Middle Eastern countries, we aimed to also recruit a narrowly defined sample of physicians with migration experience as key informants to provide insights on the regular participants, i.e. migrants without a medical professional background. While we aimed to uphold intersectional representation and transparency in our sample composition, we also had to navigate the operational challenges of conducting fieldwork. In this article, we reflect on the planning and recruitment of a key informant study, with a particular focus on balancing the ideal composition of the sample-considering power and representation - and the tension between methodological ideals and the practical realities of health research. Our KII study serves as a lens to examine the interplay between these methodological aspirations and the operational realities in the recruitment process of a key informant study.

## Background

### Conceptualizing key informants in health research

Interviews with so-called key informants originate from anthropological research, where a researcher would cultivate a relationship with one key informant over an extended period, conducting multiple interviews to understand an issue deeply [4]. While ethnographic use of KIIs embodies an embedded approach to understanding communities through long-term relationships, their application in health-related research has evolved. We refer here to ‘health research’ as inquiry that is primarily focused on understanding and improving health outcomes, often through the application of medical and public health frameworks in policy and practice, whereas anthropological research on health tends to explore health within the broader context of culture, social structures, and human behavior. In health research, engaging KIIs has become instrumental as a method for short-term knowledge production technique to rapidly gather data, often to complement quantitative information about health and health service outcomes. This reflects broader trends in ethnographic methods being adapted for speedier research, such as “rapid ethnographies” [16, 17].

In anthropological research, a core characteristic of key informants (or their role in a study) continues to be their embeddedness in a certain community which they both represent and to which they can provide insights. Furthermore, according to Marshall [16] key informants are additionally viewed “as extraordinary by those around them and usually, but not invariably, occupy a position of responsibility and influence” (p. 92). While Marshall does not further describe what constitutes this extraordinary character, a notion of authority occupied by individuals who become key informants can also be found in other seminal texts of anthropology [18–20].

In health research, key informants are primarily selected based on their professional roles or specialized knowledge of a topic [21–23]. Key informants often include healthcare providers, policymakers, scientists, community leaders, and educators who offer valuable insights into the research topic due to their positions within clinical practice, government, academia, or community organizations [8, 24]. Compared to anthropological studies in which key informants are generally embedded in their communities, key informants in health research may be ingroup members, this is however not a prerequisite criterion.

A key reason for choosing KIIs as a research method in both anthropological and health research is due to the key informants’ assumed expertise or specialized knowledge on a subject [2, 7, 8]. The term ‘key’ points to both the implied importance of the informant and their potential ability to unlock or access a previously inaccessible phenomenon [7]. ‘Informant’ indicates the researcher’s relatively unknowledgeable position compared to the informant’s insider knowledge or experience on the issue at hand [25, 26]. It also implies that the subject under research is complex and requires interpretation by a knowledgeable individual [8]. Apart from providing insights, key informants may also be chosen for their roles as representatives or ‘surrogates’ for broader groups as they are part of a community, yet also differ from it in their relationship to the research topic and researchers [4, 5]. Finally, in some contexts, interviewing certain individuals in positions of power may be necessary for political or cultural allyship, regardless of their ability to provide valuable knowledge for the research, or to later facilitate greater community access for researchers, acting as gatekeepers (or openers) to participants that are otherwise inaccessible to the researchers [5, 27].

Individuals who become key informants are often ascribed a certain authority within their respective social settings [1, 28, 29]. This authority is frequently mirrored in the way research is conducted as studies often reflect these implicit hierarchical assumptions [8]. Compared to research involving participants who are not designated as key informants, the insights and knowledge from KIIs are often perceived as “more important, accurate or objective” [7] and given greater weight when interpreting results. Given that knowledge production in qualitative research is shaped by the social and cultural contexts of informants, including their socioeconomic status, ethnicity, and education [14, 30, 31], there is a risk that perspectives of more privileged individuals may be prioritized. This can marginalize less powerful voices, raising concerns about equity and diversity in the representation of findings [3, 6, 7].

Gender dynamics offer a particularly salient example of how socially manifested authority is reflected in KII studies as research shows that studies using KIIs have resulted in a disproportionate number of male key informants [32, 33]. This is in stark contrast to the reality in both the family and community domains, as well as in the professional sector, where women continue to constitute the majority of caregivers and healthcare workers [34, 35]. Though intentional efforts to interview women equally are changing these trends [36], feminist scholars continue to highlight how men shape dominant paradigms and ways of thinking, with male experts’ opinions frequently overshadowing those of women and other marginalized groups [7, 18, 37–39]. This does not imply that women lack influence or expertise; rather, it suggests that their voices are often marginalized by the greater power hierarchies which have often led to men being recognized as experts over women [38, 40]. When combined with the methodological differences between KIIs and focus group discussions (FGDs), it becomes even more evident that KIIs may offer more opportunities for male voices to be heard in one-on-one contexts than FGDs or surveys do for women and reproduce monopolies of knowledge and authority [37, 41–43]. However, this issue is not limited to gender alone. Other dimensions of privilege, such as race, ethnicity, class, and education, similarly influence whose voices are heard and how knowledge is constructed and represented in research [3, 6]. The challenge of equitable representation in studies with KII highlights the need for selective recruitment based on an intersectional approach that addresses not just gender, but a range of intersecting identities and power dynamics for recruitment. While key informants are integral to both anthropological and health research for their unique perspectives, their selection requires careful consideration and comprehensive descriptions of recruitment processes to ensure that the inherent power dynamics and representational biases can be managed and addressed throughout the entire research process.

### Recruiting key informants for qualitative health research with an intersectional framework

Intersectionality is rooted in Black feminism but has increasingly become a critical theory across disciplines including health research. Intersectionality describes a framework for understanding multiple, intersecting forms of oppression within a matrix of domination rather than observing discrimination as distinct or independent phenomena [12, 13, 44]. Intersectionality hence emphasizes the interconnectedness of multiple socially constructed categories (e.g., ethnicity, gender, migration status, class) and the linked systems of power and inequality that lend more or less privilege to holders of certain social identities [45]. Condensed, intersectionality is based on the assumption that (a) all individuals possess or embody multiple intersecting categories; (b) each identity includes a dimension of power or oppression; (c) these categories are shaped by socio-cultural contexts and are therefore not universal, and; (d) individuals can therefore belong to both oppressed and privileged groups in different contexts [12, 13, 46, 47].

Since its evolution, intersectionality has been employed across various disciplines, including health research and can be particularly relevant in reflections on study participant recruitment processes. Authors have pointed out that studying so-called “hot samples” ( [48], p. 293) with known intersecting social categories, e.g., Black lesbians, low-income or homeless people, has been the most dominant approach when applying an intersectional lens in recruitment [47–49]. This overreliance on known categories of privilege and oppression creates epistemological issues. First, even with the intent to be loyal and sympathetic to minority groups, such research risks perpetuating othering processes, hence portraying such groups as fundamentally different from more privileged study participants [48]. On the one hand, reflecting on social categories may serve as a tool for selecting marginalized participants and making oppression visible. Yet power should not only be conceived in terms of combating oppression in a one-directional sense. Working within power dynamics can also reconfigure power relations, intertwine social categories and analyze how people move from positions of lesser to greater privilege or vice versa [50]. For instance, when recruiting key informants who often hold more power or privilege in certain social settings but may also share one or several other social categories with those of other study participants, it is important to consider how to weigh their insights and how their elevated status may inadvertently overshadow the voices of less privileged participants [48, 50–52]. These epistemological considerations are particularly important given that health research is an applied rather than theoretical field, and they can therefore have significant and material impacts, such as inadvertently contributing to stigmatization of certain groups or shaping access to (health) resources.

A second critical question concerns the number of categories to consider, as including too few categories risks oversimplification, potentially overlooking important nuances in how identities intersect. Conversely, including too many categories can make the recruitment unwieldy, making it challenging to maintain focus on the most relevant intersections within small qualitative samples. This necessitates the strategic selection of a limited number of categories or establishing anchor points for analysis [47]. Moreover, in the exploratory nature of qualitative research, certain unmarked categories may only emerge during the analysis phase, highlighting the dynamic and fluid understanding of intersecting social categories that cannot always be fully anticipated during the recruitment process.

### Recruiting an ‘ideal’ sample with the practical realities of recruitment

While striving for a carefully composed, ‘ideal’ sample based on reflections of intersectionality for KII in research, the sample representation is inherently dependent on whom researchers are able to reach: hence how balancing these methodological ideals interacts with practical challenges in real-world recruitment. In qualitative research, the recruitment is often challenging and exploratory, as the process cannot be fully predetermined before fieldwork begins [53]. Not every social category can be adequately captured, not all participants may be willing or able to disclose certain aspects of their identity and the intersection of multiple desired social categories narrowly defines a sample, increasing difficulties in recruiting desired participants. This means that even with clear goals, the recruitment process often involves making subjective decisions about whom to approach, how to gain access to them and making compromises if the ideal sample cannot be reached. As a result, the planning and recruitment needs to be flexible and responsive to the realities of who is actually accessible to researchers in the field, including cultural, legal and regulatory norms and alongside considerations about representation and power in knowledge production. This consideration brings attention to the importance of transparency in the planning stages, including the power dynamics at play, the recruitment procedures (such as the use of incentives and specific approaches), and the researcher’s positionality, who are themselves socially situated and whose social positions might influence recruitment procedures.

The objectives of this paper are to (1) describe our considerations in composing an ideal sample for a KII study within our research project on migrants’ access to cancer screening and prevention, with particular attention to issues of power and representation, and (2) reflect on the recruitment strategies, challenges, and successes in gaining access to this sample, highlighting how these dynamics influenced the research process and outcomes.

## Methods

### About the KII study

Over the past decade, Germany’s demographic landscape has shifted following the admission of over one million refugees and migrants from Middle Eastern countries (i.e., Syria, Iraq, Iran, Palestine, and Afghanistan) and the Ukraine [54, 55]. Existing research has focused on larger migrant communities with a longer migration history, especially those with Turkish and Russian backgrounds [56, 57]. Studies among migrants with a relatively recent migration history in Germany are still scant and fragmented [58–60]. Hence, our research specifically targets first-generation migrants from the Middle East, based on certain similarities in healthcare system structures, cultural and religious backgrounds that influence health beliefs, behaviors, and access to healthcare services, in ways that differ from other migrant groups. For instance, some studies show that healthcare needs of newly arrived migrants tend to focus on immediate needs, and less on taking part in cancer screening measures, e.g., mammography, pap smear or prostate cancer screening [60–62].

Given the lack of migration- and culture-specific data on cancer screening participation among these populations, the KIKK project (German abbreviation for culture-sensitive health information for cancer prevention and screening) aims to explore factors influencing (non-)participation in cancer screening among first-generation Middle Eastern migrants. One component of this research project involves a KII study with physicians with specialist training in clinical disciplines relevant to cancer prevention and screening, such as urology, gynecology and dermatology. Oncologists and other disciplines focused primarily on patients with an existing cancer diagnosis were excluded.

### Procedures

#### Theoretical composition of the KII sample

We applied selective recruitment based on an intersectional approach to consider the social categories that appear relevant to understanding the dynamics of cancer screening and prevention among Middle Eastern migrants in Germany, as well as within the KII sample. We defined the social categories gender, migration background, cultural and linguistic background, and healthcare setting as key to our research (see Table 1 for an overview). Our approach to KIIs was purposefully designed to capture insights from physicians serving as key informants as these individuals combine both cultural insider perspectives and professional expertise. Unlike a general stratified sample for qualitative interviews, our selection of key informants was intended to contextualize patient experiences of migrants within the healthcare system, using these key informants who can reflect both insider and outsider perspectives. Since the research project is in its initial stages, we have considered these social categories separately during the recruitment process and will focus on exploring how these categories intersect and influence one another during the analysis. To facilitate this, we will intentionally seek participants who embody multiple characteristics across these categories, allowing us to examine the complex ways in which these intersecting identities shape healthcare experiences and outcomes.

Our goal was to recruit physicians who embody both insider and outsider perspectives. Hence, we exclusively sought to recruit physicians with a *migrant background* who are themselves first- or second-generation migrants from Middle Eastern countries and who can relate to the populations in question due to their cultural and linguistic ties. At the same time, in their professional roles, these physicians can also be considered outsiders from migrant communities, possessing specialized knowledge of the medical system in Germany, which positions them uniquely between their communities and the broader healthcare infrastructure.

Diversity in *professional disciplines* related to cancer screening is essential to our KII study, given the varying levels of invasiveness associated with different cancer screening procedures. For example, a pap smear, which is critical for detecting cervical cancer, may be perceived differently by women from Muslim-shaped backgrounds, who may associate it with the loss of virginity or other gender norms around modesty. Similarly, screening procedures that require undressing may be influenced by beliefs about gender and body exposure. By including physicians from a range of disciplines involved in cancer screening, such as gynecology, urology, general practice, or dermatology, we sought to understand how these professional perspectives intersect with cultural and gender norms to influence healthcare delivery.

We deemed *gender* a critical axis in our sample composition. In both the general population and the healthcare sector, gender continues to influence access to resources, decision-making processes, and representation in leadership roles. Recognizing that previous studies have often disproportionately featured male key informants as experts in KIIs, we sought to ensure gender balance in our sample. Moreover, gender itself is a highly relevant category in cancer screening, as the healthcare service offers differ for men and women. For instance, early detection screening for breast and cervical cancers are targeted at women, while there is a focus on men for prostate cancer screening, reflecting gender-specific examinations. Including a focus on gender in the KII will allow us to explore how gendered experiences and expectations influence cancer screening practices and the provision of care across different patient groups.

*Cultural and linguistic background* plays a role in both the health-seeking behaviors of migrant patients and the practice of physicians. For non-medical participants, culturally shaped health beliefs and language proficiency can impact access to cancer screening services. With our research focus on migrants from Middle Eastern countries, we aimed to include physicians proficient in languages such as Farsi, Dari, Pashto, Arabic, or Kurmanji, who share cultural and linguistic backgrounds with the migrant communities of interest. We assumed that these physicians’ cultural backgrounds influence their understanding of health beliefs and practices, which may differ substantially from those of their German-trained counterparts. For instance, different cultural norms around different communication styles or participation in healthcare decision-making can affect how cancer screening services are perceived and utilized. By including physicians who are attuned to these cultural nuances, we aimed to explore how health beliefs and knowledge about care delivery intersect with professional practice.

Acknowledging that while the ‘*setting*’ in which healthcare is provided, whether in hospitals or ambulatory/outpatient care, is not a traditional intersectional category, it may also intersect with power dynamics, shaping healthcare practices and access to cancer screening in the German context. Physicians working in hospital settings may have different perspectives compared to those in ambulatory or outpatient practices, particularly in how they manage patient flow, waiting periods, and access to screening appointments. The German healthcare system, with its emphasis on outpatient care and longer waiting times for non-urgent procedures, may be unfamiliar to recent migrants, who may come from countries with different healthcare delivery models. By including physicians from both hospital and outpatient settings, we aimed to capture how these structural differences intersect with other social categories to shape healthcare-seeking behaviors and the provision of cancer screening services.

**Table 1:** Overview of recruitment criteria

Recruitment criteria	Definition
Gender	Gender balance, with a focus on the representation of women
Professional discipline	Physicians from different medical disciplines related to cancer screening, e.g., urology, dermatology
Migration background	First- or second-generation physician migrants from different Middle Eastern countries, e.g., Iran, Syria, Afghanistan
Cultural and linguistic background	Physicians proficient in languages spoken in the Middle East, e.g., Farsi, Dari, Pashto
Setting	Physicians working in different inpatient and outpatient healthcare settings

#### Operationalizing recruitment criteria in reality

In operationalizing our recruitment criteria for the KII study, we determined that a professional discipline related to cancer screening and a migrant background from the Middle East were essential for inclusion. Physicians who did not meet these criteria would be excluded from the KII study, as their perspectives would not align with the specific focus of our research on the intersection of migration and cancer prevention. To build a sample that reflects the diversity across the five key social categories - gender, migration background, cultural and linguistic background, healthcare setting, and professional discipline - we employed an iterative approach. This approach involved continuously assessing who responded to our recruitment outreach and strategically targeting specific groups to ensure a balanced, yet maximally diverse, sample. By adapting our recruitment efforts based on the demographic and professional profiles of early respondents, we aimed to capture a wide range of experiences and insights that align with our intersectional framework.

#### Exploring access strategies

To effectively recruit participants, we employed both direct and indirect recruitment strategies, tailored to identify and engage physicians who met our inclusion criteria.

Our direct recruitment strategy involved identifying suitable physicians and contacting them without intermediaries. We utilized the online systems of the Association for Statutory Health Insurance Physicians (ASHIP) across Germany’s federal states. These systems allowed us to filter for registered physicians by geographical location, medical specialty, and consultation languages. By focusing on relevant specialties such as general and internal medicine, gynecology, gastroenterology and urology, and combining these with language requirements (e.g., Arabic, Turkish, Kurmanji, Dari, Pashto, Farsi), we would be able to directly reach out to potential participants who fit our study’s criteria. For each potential participant, we conducted a review of their institutional background, determining the specific healthcare setting in which they worked (e.g., hospital, outpatient clinic). When available, we also examined practice websites, where profile pictures often helped us ascertain the gender of the physicians.

For indirect recruitment, we incorporated snowball sampling and leveraged multipliers to disseminate information about our KII study. Through snowball sampling, we asked already recruited interviewees to recommend colleagues who met our recruitment criteria, which successfully led to the inclusion of additional participants. Additionally, we engaged multipliers - individuals or platforms with access to our target population. This included mentions in a podcast by a Turkish-descent podcaster, posts in a large WhatsApp group for foreign doctors in Germany by one if its members, and emails to hospital managers requesting them to forward our recruitment call to staff.

#### Preparing and testing recruitment materials

To initiate our recruitment process, we prepared a comprehensive email text that outlined all relevant information regarding the study’s purpose, the role of participants, and logistical details such as interview length, the amount of the incentive (70 €) and scheduling flexibility (see attachment). This strategy was tested by sending it to 50 physicians identified through the ASHIP database, but no responses were received.

To improve our strategy, we consulted four physicians within our professional networks, who suggested changes to make key details - such as our university affiliation, the principal investigator’s doctoral title, interview length, scheduling flexibility, and financial compensation - more prominent. They approved the suggested amount of 70 €, considering it sufficient as an incentive for study participation, yet lower than a physician’s hourly salary. They also recommended sending emails on Wednesdays when physicians are more likely to check their emails due to administrative work. We further explored using phone calls and faxes as additional outreach methods to increase our chances of contact, but these proved ineffective. After 99 unsuccessful phone calls and limited responses to faxes, we discontinued these methods and focused on follow-up emails after an initial email. These follow-ups, sent within a week, emphasized our previous outreach and the importance of their participation.

For indirect recruitment, we used the revised email text for networks and adapted it into a more concise version for platforms like WhatsApp groups and podcasts, ensuring the essential details were communicated effectively.

#### Screening and recruitment

Whenever we received a response to any of our outreach efforts, we promptly screened the potential participants against our established inclusion criteria. While we adhered to the core criteria, we also made adjustments to include participants who, though not originally from the Middle East, had acquired relevant language skills, as well as physicians who had transitioned to other specialties, such as two participants now working in neurology and psychiatry, after having been involved in cancer screening as general practitioners.

Initially, our direct recruitment efforts yielded responses predominantly from women working in disciplines such as gynecology and general practice. Recognizing this imbalance, we specifically redirected our searches in the database to target other disciplines, such as urology, gastroenterology and dermatology, and slightly adjusted our outreach efforts to increase the likelihood of recruiting male participants. If respondents met these adjusted requirements, we provided them with additional study information, including details on data protection and a consent form. Upon receiving their consent, we scheduled interviews via video conference, ensuring that all logistical details were clear and convenient for the participants.

#### Positionality of the research team

Our research team is composed entirely of women, totaling five members. This includes two research associates, one of whom is the PI, two student assistants, and a professor. All team members possess tertiary educational backgrounds related to public health. Among us, four members do not have a migration background, while one member is a first-generation migrant. In addition, the PI has lived abroad and has extensive experience in international research projects. While our collective academic background equips us with the necessary tools for rigorous research, we remain mindful of the potential limitations and biases that our positionality may bring, particularly in understanding the experiences of migrant populations within the German healthcare system. Our all-female composition brings a unique sensitivity to gender-related issues in healthcare, which can enhance our understanding of women’s experiences but may pose a risk of bias on male perspectives in our analysis and interpretation.

## Results

### Response rates via direct and indirect recruitment strategies

We employed direct and indirect strategies in our recruitment efforts.

**Fig. 1:**
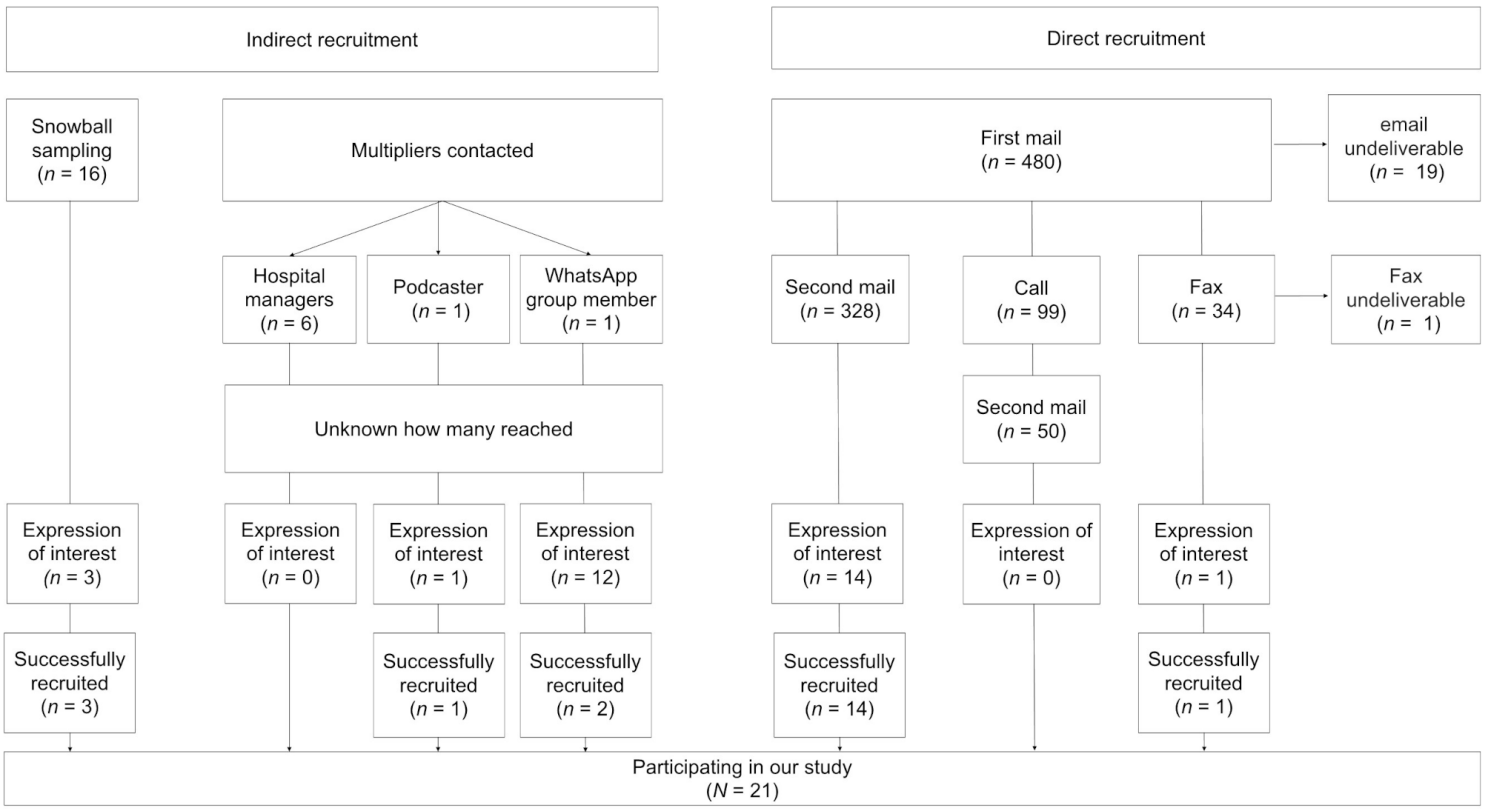
Overview of recruitment strategies

*Direct recruitment*. In total, we contacted 480 physicians directly (with *n* = 20 undeliverable emails/fax and *n* = 460 non-responders) (see Fig. 1 for an overview of our recruitment strategies). Sending two consecutive emails to 328 practices and institutional addresses of physicians resulted in 14 positive responses of whom all were included in the KII study due to meeting our selection criteria; one person responded after an initial email, followed by a fax; none after phone calls. Recruitment took place over the course of eight weeks and we updated our database based on the responses we received. General practitioners and gynecologists were the first to respond, whereas we did not receive any responses from urologists and gastroenterologists for six weeks and therefore sent out a considerable number of emails to recruit from this clinical subset. Despite extensive searches and outreach with 170 emails alone to this subset, we were only able to recruit two urologists and one gastroenterologist.

*Indirect recruitment*. Indirect recruitment subsumes two strategies, namely snowball sampling and outreach via multipliers. Results from reaching out to multipliers were mixed in their quality and quantity received (hospital managers: *n* = 0, podcaster: *n* = 1, WhatsApp group: *n* = 12). Despite the greater rate of response, respondents from the WhatsApp group shared many similarities in their professional and sociodemographic data: most came from Turkey, were women and had little to no work experience in Germany. Overall, only three respondents via the multiplier strategy were included in the KII study. Snowball sampling yielded three expressions of interest of whom all three were included, resulting in a total of six participants via indirect recruitment.

### Recruited sample

We included 21 physicians with diverse social categories (see Table 1 for characteristics of our sample). The sample includes slightly more women (57%) and physicians working in ambulant/outpatient care settings (60%). While all participants were first- or second-generation migrants, 18 originated from a Middle Eastern country and three originated from other countries and had acquired language skills from the Middle Eastern region during their studies or work in that region. At the time of the KII study, all but two participants were working in a cancer screening-related discipline, two had done so in the past, but had proceeded on to further training in a different medical discipline.

**Table 2:** Professional and sociodemographic data of recruited physicians (*N* = 21)

Characteristics		*n*	%
**Gender**			
	Female	12	57
	Male	9	43
**Professional discipline**			
	Gynecology	5	23
	General practice	5	23
	Internal medicine	3	14
	Dermatology	2	10
	Urology	2	10
	Pneumology	1	5
	Gastroenterology	1	5
	Neurology	1	5
	Psychiatry	1	5
**Migration background (first and second generation)**			
	Syria	5	23
	Iran	4	19
	Turkey	4	19
	Palestine	2	10
	Iraq	1	5
	Afghanistan	1	5
	Jordania	1	5
	Azerbaijan	1	5
	Romania	1	5
	Ukraine	1	5
**Language skills from the Middle Eastern region (more than one possible)** ^**1**^			
	Arabic	11	52
	Turkish	6	29
	Persian/Farsi	4	19
	Kurdish (Kurmanji)	3	14
	Pashto	1	5
	Hebrew	1	5
	Dari	1	5
**Clinical setting/place of work**			
	Outpatient/ambulant	13	62
	Inpatient/hospital	8	38

Note 1. One participant spoke three languages from the Middle East, four spoke two

## Discussion

This KII study aimed to explore the challenges and strategies involved in recruiting a diverse sample of key informants - specifically, physicians with a migration background from Middle Eastern countries who are involved in cancer screening and prevention in Germany. By applying an intersectional approach to the recruitment strategy and being transparent about our selection criteria, the KII study sought to address issues of power, representation, and inclusion in the sample composition, while balancing these methodological ideals with the operational realities of fieldwork [2, 7].

Recruiting physicians as participants in research is notoriously challenging, with studies consistently highlighting obstacles such as time constraints, lack of perceived relevance to clinical practice, limited familiarity with research methodologies, and over-saturation of research requests [21, 22]. When applying narrowly defined recruitment criteria, such as physicians with specific language skills, clinical specializations, and settings, the task becomes even more daunting. Our experience underscores this reality, demonstrating that even though physicians are key informants with expertise, they remain a difficult group to engage, particularly when the recruitment criteria are highly specific and, as in our case, physicians were not native speakers of the language the interview was conducted in. This could have lowered response rates as two participants explained during data collection.

In this, our experience highlights the importance of an iterative approach to recruitment as it allowed us to continuously assess, reflect and refine our strategies to ensure our desired, equitable representation and cope with the challenges of recruiting this difficult-to-reach sample [6]. For instance, in our recruitment efforts, the direct recruitment channel emerged as more effective, particularly after refining our initial email approach. Once optimized, this method allowed us to send out more targeted, time-efficient communications, which proved essential in reaching our goal. Our ability to filter and select physicians from the ASHIP database, combined with persistent follow-up efforts, resulted in successful recruitment, highlighting the value of adaptability in real-world settings. Offering a compensation of 70 € was likely crucial for successful recruitment, particularly among busy professionals like physicians, as it provided a sufficient incentive while remaining within an ethical range that upheld the credibility of the research. However, this also highlights a dilemma: while such compensation may be deemed appropriate and necessary for key informants, such as physicians, it would be perceived as undue influence if offered to other participant groups, such as patients. Nonetheless, without a sufficient incentive, we believe it would have been less likely to achieve the necessary sample size. Moreover, after initially low response rates from male physicians and certain specialties, we intensified our efforts in those areas, extending our search within the database. However, the process was not without challenges. Outdated or incorrect contact information in the ASHIP’s online systems led to undeliverable emails, and the effectiveness of follow-up communications varied. For instance, we found that contacting potential participants on Wednesdays - when practice hours are shorter - yielded better responses. Conversely, indirect recruitment methods, such as using multipliers or snowball sampling, had lower success rates than anticipated. While these approaches did yield respondents, they were homogenous and we had little control over the characteristics of respondents, consequently declining the majority for the sake of a more heterogenous sample composition.

It is important to recognize that our recruitment strategies represent just an initial step in addressing the broader question our article raises: Whose voices and knowledge are being prioritized or amplified, and whose are marginalized in research processes? While we have strived to foster a diverse key informant sample through selective recruitment informed by intersectionality, the effects of these efforts will only be revealed during data analysis. It is during this phase that the actual distribution of perspectives, and their intersectional dimensions, will be critically examined, making clear how different social categories - such as gender, ethnicity, and professional status - interact [12, 13]. This underscores the necessity for ongoing reflexivity throughout the entire research process and across different methods [6, 30].

The role of key informants in this study - and in qualitative research more broadly - demands careful scrutiny, particularly when their insights are compared with those of regular participants who may not possess the same level of professional expertise yet different, insights based on their lived realities [3, 6, 9]. Key informants, by virtue of their positions in society, often hold a degree of power that can shape the narratives that emerge from the research, if not reflected upon [2, 7]. In studies that involve multiple data sources and types such as KIIs and FGDs, the question of whether all data is or should be considered equal becomes particularly relevant [10, 63]. The insights provided by key informants, while valuable, should be carefully balanced against those of regular participants to avoid reinforcing existing power dynamics and perpetuating disparities in representation [6, 7]. Hence, reflexivity, transparency and methodological rigor from recruitment to data analysis and presentation are required. These principles contribute to ensuring that studies remain true to their goal of amplifying diverse voices while maintaining a careful balance between professional or societal authority and lived experience.

## Limitations

This KII study has several limitations. The sample faced selection bias, particularly in the overrepresentation of certain nationalities (e.g., Syria, Turkey, and Iran) and women, while other groups (e.g., Afghanistan and Iraq) were underrepresented. However, this selection of countries reflects the demographic distribution of foreign-trained physicians in Germany, where Syrian, Turkish, and Iranian doctors constitute some of the largest groups of migrant healthcare professionals [64]. A self-selection bias is also likely as non-native speakers in German or English, in which interviews were conducted, might have felt hesitant to participate in our study. This highlights how linguistic barriers can influence the scope of perspectives included in research and underscores the importance of accommodating various participant needs, such as linguistic and cultural diversity, to allow for equitable access to participation.

Additionally, the unique use of the ASHIP database, specific to the German healthcare system, limits the generalizability of our findings to other contexts. The resource-intensive nature of our direct recruitment strategy, requiring substantial time and effort in filtering databases, sending out emails, and making phone calls, emphasizes the need for careful planning and allocation of resources in future studies. Finally, the true diversity and representativeness of our sample will only be fully understood during the analysis phase, where the interplay of various social categories can be thoroughly examined. In this way, our limitations highlight the practical realities of translating theoretical ideals into applied research and emphasize the role of adaptability and reflection in overcoming recruitment barriers.

## Conclusion

This KII study underscores the tension between methodological ideals and the operational realities of recruiting a diverse and carefully composed sample of key informants in health research. While our intersectional approach aimed to ensure equitable representation by carefully considering power dynamics and refining our recruitment strategies, the challenges of real-world fieldwork - such as the difficulty of engaging busy physicians with specific criteria - required a balance between ideal recruitment practices and practical adaptability. The recruitment process highlighted the importance of being flexible and persistent in overcoming barriers, while also remaining critically aware of how these operational choices influence which voices are amplified or marginalized. Ultimately, this work emphasizes the need for ongoing reflexivity in research to navigate the complexities of achieving both methodological rigor and practical feasibility, ensuring that the diverse realities of participant experiences are accurately represented in studies’ findings.

## Electronic supplementary material

Below is the link to the electronic supplementary material.

Supplementary Material 1

## Data Availability

No datasets were generated or analysed during the current study.

## Abbreviations

ASHIP: Association for Statutory Health Insurance Physicians
FGD: Focus group discussions
KII: Key Informant Interviews
