# Supplementary material for: Balancing between reality, ideality, and equity: critical reflections from recruiting key informants for qualitative health research
Source: BMC Med Res Methodol. 2024 Nov 12;24:276. doi: 10.1186/s12874-024-02403-2 (PMC11555920; doi:10.1186/s12874-024-02403-2)
Supplement: Supplementary file 1 — Supplementary Material 1 [file 12874_2024_2403_MOESM1_ESM.pdf]

Supplementary material

First and second email to physicians

Subject: Interview Study (University of Bielefeld): Invitation to Participate in an Online Interview

Email 1: Initial email

Dear Dr. X,

While searching for doctors who offer consultations in foreign languages, we came across your profile in the database of the Association for Statutory Health Insurance Physicians (ASHIP).

We are researchers from the University of Bielefeld, School of Public Health, conducting a project funded by the German Cancer Aid. The project aims to develop strategies to improve access to cancer screening within the German healthcare system for people with migration backgrounds from the Middle East.

The interview will take place via a video conferencing tool and will last a maximum of 60 minutes. We will accommodate your schedule and offer you €70 for your participation.

About the Study

The study focuses on exploring access opportunities and barriers for people with migration backgrounds, particularly the first generation of immigrants from the Middle East, in obtaining information about cancer prevention and early detection. A special focus is on identifying and understanding the specific cultural, linguistic, and other sociodemographic needs as well as key sources of information for people from this heterogeneous population group.

To develop appropriate intervention strategies, we would like to discuss the following topics with you:

- Your experiences in medical consultation with migrants (from the Middle East)
- Access and barriers in the self-directed engagement with health information relevant to the German healthcare system by the target group
- Important multipliers and networks for health information

We would be very grateful if you could share your experiences with us and help us develop culturally sensitive health information on cancer prevention and early detection. If you are unable to participate but can recommend another doctor, we would appreciate that as well.

How to Participate in the Study

Please respond to this email with three proposed dates for the interview and we will get back to you. Attached, you will find the information for participants, including the data protection declaration.

We look forward to your response.

Best regards,

Research team

Email 2: Follow-up

Dear Dr. X,

we contacted you last week (see the email below) and also tried to reach you by phone, but unfortunately, could not reach you.

We understand that, due to cold season, other infection waves, and staff shortages, you are extremely busy. Nevertheless, we would like to encourage you to participate in our study. We are happy to schedule appointments with you, even outside of regular working hours, if that would be more convenient.

If it would be easier for you to participate at the beginning of next year, that is also an option.

A reminder about the study:

We – researchers from the University of Bielefeld, School of Public Health – are conducting a project funded by the German Cancer Aid, which aims to develop strategies to improve access to the German healthcare system and health information for people with migration backgrounds from the Middle East.

The interview will take place via a video conferencing tool and will last a maximum of 60 minutes. You will receive €70 for your participation. We are flexible in scheduling according to your availability.

If you are unable to participate at this time but can recommend a colleague with a similar background who might be interested, we would greatly appreciate it if you could share the study information with them. Please feel free to let us know if you need any further information.

We look forward to hearing from you and remain with kind regards,

Research team

Interview guide: medical personnel with migration experience

Topics

1) Pre-Interview: Sociodemographic data
2) Characterization of contact groups
3) Doctor-patient communication
4) Clients' autodidactic engagement with health information
5) Dual role of migration background and medical professional in German healthcare system

1) Pre-Interview: Sociodemographic data

- Before we dive deep into the subject, I have some personal questions for you. Can you briefly introduce yourself and talk about your specialty, how long you have been working in it, and where you received your training?
    - How many years of professional experience do you have in this field?
    - How many of those in Germany/in the German healthcare system?
- Were you born in Germany?

  - If not, how long have you been living in Germany?
- To what extent do you deal with cancer, cancer prevention, and early detection in your daily professional life?
- With which country/countries or culture do you have a special connection?
- What languages do you speak, and in which do you offer medical consultations?
  - Are there differences in the language skills you possess?

2) Characterization of contact groups/clients

- We selected you for this interview because you offer consultations in language X. Do patients come to your practice specifically for this reason?
  - If yes, can you roughly estimate what proportion of these individuals, who come to your practice because of the language offering, make up among all your patients?
  - Do you also know from what area these people come to you? And how they find out about you?
- The group of people with a "migration background" is very heterogeneous – can you describe more closely whom you deal with in your professional everyday life?
- Thinking about these people from your everyday life – how do you assess them in terms of:
  - Their knowledge about the German healthcare system?
  - Their trust in doctors in Germany?
  - Their understanding or knowledge of prevention and early detection?
  - How do these factors differ from those of other patients?

3) Doctor-patient communication

- We would now like to know how you experience conversations with people of language or culture X. What are, in your experience, specific challenges in conversations with people with a migration background?
  - What makes the conversations difficult? (prompt if necessary linguistic differences (dialects, etc.), differences in health understanding in the country of origin, hierarchies between doctor and patient)
  - How do you deal with challenges?
- What do you find helpful in conversations with migrants?
  - What are your strategies, for example, what helps you build trust with your patients?
- Have you ever used interpreters for doctor-patient communication?
  - If yes, can you talk about it? Were they professional or lay translators?
- To what extent does language mediation affect the dynamics of the conversation?
  - Can you think of an example where language mediation did not work at all?
  - If so, what were the reasons?
- What role do relatives play as language mediators?
  - What have you experienced in language mediation with and through relatives?
- Have you also used technical tools like translation software?

4) Clients' autodidactic engagement with health information

- People can inform themselves about health through a variety of channels, much of which takes place outside of clinical settings. The quality and type of health information can vary greatly. Could you briefly describe from your perspective what makes "good" health information?
- How can we inform people with a migration background about prevention and early detection and sensitize them to it?

  - Which channels are suitable?
  - What increases their credibility?
  - Who could serve as mediators or bridges between the individuals and the healthcare system?
  - How could health information for people with a migration background be designed so that 1) it is found and 2) understood?
  - Conversely asked: how should health information not be?
- Besides the quality of health information, the personal situation of the individuals receiving the information also plays a role. Can you describe the "prototype" (make quotation marks with fingers) of a person with a migration background who has good access to health information?
  - What is the sociodemography of this person?
  - What are the differences between feature X and Y (e.g., male versus female person -> adjust according to the interviewee's statements) and this person's informedness?

5) Dual role of migration background and medical professional in German healthcare system

- Due to your special connection to country/culture X and your language skills (adjust), you have certainly had personal experiences with the German healthcare system. What have you learned from your own migration history about accessing health information in the German healthcare system for your work/for dealing with your patients?
- What challenges have you faced when it came to finding or understanding health information (whether personally or for a close person)?
  - If you have encountered challenges, how have you dealt with these challenges?
  - Dream for a moment – what would have helped you in this situation, what would you have wished for?
- How do you see your role/perspective as a professional with a migration background in the German healthcare system?
  - Do you have a special role compared to professionals without a migration background?
  - What should your colleagues without a migration background know about conveying health information to patients with a migration background?
  - What should authorities/healthcare structures know and take into account?

- I have finished my questions so far. Is there anything else you would like to add to this conversation?
